# Trends in Primary Antibiotic Resistance in *H. pylori* Strains Isolated in Italy between 2009 and 2019

**DOI:** 10.3390/antibiotics9010026

**Published:** 2020-01-13

**Authors:** Ilaria Maria Saracino, Giulia Fiorini, Angelo Zullo, Matteo Pavoni, Laura Saccomanno, Dino Vaira

**Affiliations:** 1 Department of Surgical and Medical Sciences, University of Bologna, 40126 Bologna, Italy; saracinoilariamaria@gmail.com (I.M.S.); giulia.fiorini@fastwebnet.it (G.F.); matteo.pavoni@studio.unibo.it (M.P.); laura.saccomanno@studio.unibo.it (L.S.); 2 Gastroenterology Unit, Nuovo Regina Margherita Hospital, 00153 Rome, Italy; zullo66@gmail.com

**Keywords:** *H. pylori*, antibiotic resistance, first line therapy

## Abstract

Background and aims: the increasing prevalence of strains resistant to antimicrobial agents is a critical issue for the management of *Helicobacter pylori* infection. This study aimed to evaluate, in Italian naïve patients, *H. pylori* antibiotic resistance trends and their potential predictive factors during the last decade. Methods: consecutive Italian naïve *H. pylori* positive patients, referred from General Practitioners to our Unit from January 2009 to January 2019 to perform an upper gastrointestinal endoscopy (UGIE), were considered. Each patient underwent ^13^C-urea breath test (^13^C-UBT) and UGIE with multiple biopsies to perform rapid urease test (RUT), culture/susceptibility test (vs. clarithromycin, metronidazole, levofloxacin), and histopathological examination. *H. pylori* status was assessed through CRM (composite reference method: at least two tests positive or only culture positive). Results: between 2009 and 2014, 1763 patients were diagnosed as *H. pylori* positive, 907 were naïve with antibiogram available. Between 2015 and 2019, 1415 patients were diagnosed as *H. pylori* positive, antibiotic susceptibility test was available in 739 naïve patients. *H. pylori* primary antibiotic resistance rates in the first and second five-year period were, respectively, clarithromycin 30.2% (95% CI 27.2–33.3), 37.8% (95% CI 34.2–41.4); metronidazole 33.3% (95% CI 30.2–36.5), 33.6% (95% CI 30.2–37.1); levofloxacin 25.6% (95% CI 22.8–28.5), 33.8% (95% CI 37.4–47.4), double resistance clarithromycin-metronidazole 18.9% (95% CI 16.4–21.6), 20.7% (95% CI 17.8–23.8). The increase of the resistance rates to clarithromycin and levofloxacin in naïve patients was statistically significant (*p* < 0.05). Although eradication rates for sequential therapy in the 10 years considered were 93.4% (95% CI 92–94.6) and 87.5% (95% CI 85.7–89) at per-protocol (PP) and intention-to-treat (ITT) analysis, respectively, they showed a significant decrease in the second five-year period. Conclusions: this data highlights an increase in primary *H. pylori* antibiotic resistance and strongly suggests the importance of drug susceptibility testing also in naïve patients.

## 1. Introduction

*Helicobacter pylori* infection is correlated to upper gastrointestinal diseases such as peptic ulcers, gastric mucosa associated lymphoid tissue lymphoma (MALT), and gastric cancer [1]. Antibiotic resistance is an increasing problem for eradication therapies, the trending abuse of antibiotics is probably the cause of this issue. The selective pressure of the antibiotic intake causes modification in the genetic pattern of *H. pylori* that stays stable generation after generation [2]. Clarithromycin is a key antibiotic in *H. pylori* eradication regimens, it is a macrolide and inhibits protein synthesis by binding to the 23S rRNA component of the 50S subunit of the ribosome. Clarithromycin resistance is due to several point mutations in 23S rRNA gene; A2143G, A2142G, and A2142C represent > 90% of the observed mutations with confirmed clinical relevance [3,4]. Metronidazole is also involved in the eradication of the bacterium. It is a 5-nitroimidazole activated by *H. pylori* nitroreductase enzyme. In particular, the inactivation of rdxA (encodes an oxygen-insensitive NADPH nitroreductase) and frxA (encodes a NADPH flavin oxidoreductase) genes is highly associated with metronidazole resistance in *H. pylori* [5]. Levofloxacin, a fluoroquinolone used in rescue therapeutic regimens, interacts with type II topoisomerases preventing the unwinding of DNA and DNA replication. Mutation in GyrA or GyrB genes are linked to levofloxacin resistance in *H. pylori* [6]. In 2017 the World Health Organization published a list of antibiotic resistant “priority pathogens”, a catalogue of bacteria that pose the greatest threat to human health, and clarithromycin resistant *H. pylori* was categorized as a high-priority bacterium [7]. Resistance to fluoroquinolones can also impair the efficacy of eradication regimens [8,9,10], whereas resistance to nitroimidazole can be partially overcome in vivo when used in quadruple therapies [11]. Antibiotic agents used for *H. pylori* eradication are also widely and improperly used to treat other infections [7,8,12]. For this reason, antibiotic resistance develops continuously, so it is very important to carry out periodic assessments of *H. pylori* primary antibiotic resistance rates and to monitor the efficacy of first line treatments [13,14,15,16], thus helping clinicians in selecting the most appropriate therapy in their setting [17]. Current Italian guidelines suggest sequential or Pylera^®^ therapy as first line treatments [8,9,18]. Sequential therapy consists of 5 days of a dual therapy with PPI (proton pump inhibitor) and amoxicillin both twice a day followed by 5 days of a triple therapy with PPI, clarithromycin, and metronidazole all twice a day. Pylera^®^ therapy consists of three Pylera^®^ tablets four times a day with PPI twice a day for 10 days. The aim of our study was therefore to evaluate, in Italian naïve *H. pylori* positive patients: (1) resistance rates trends for clarithromycin, metronidazole and levofloxacin over two five-year periods, from 2009 to 2014 vs. 2015 to 2019; (2) which factors are potentially correlated with primary *H. pylori* drug resistance; (3) the effectiveness of sequential therapy.

## 2. Results

**Figure 1:**
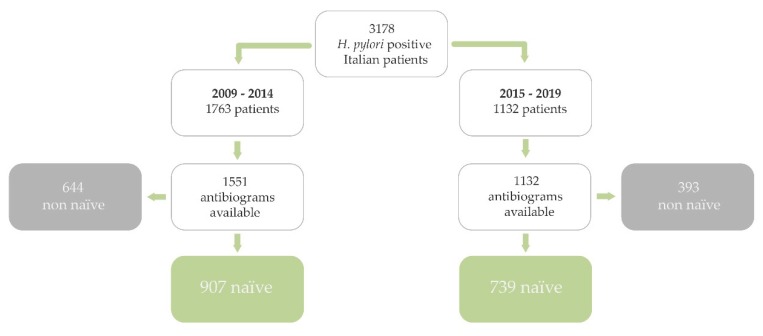
Population flow chart. Only *Helicobacter pylori* positive naive patients born in Italy were considered.

A total of 3178 Italian patients were infected, 1646 were naïve with antibiogram available (M/F: 646/1000; median age: 51 years, range 18–85 years). A total of 1763 Italian *H. pylori* positive patients underwent endoscopy between 2009 and 2014, and antibiotic susceptibility test was available in 1551, 907 were naïve; whilst 1415 Italian *H. pylori* positive patients underwent endoscopy between 2015 and 2019, and antibiogram was available in 1132, 739 were naïve (Figure 1).

Population features are reported in Table 1a,b and Table 2.

**Figure 2:**
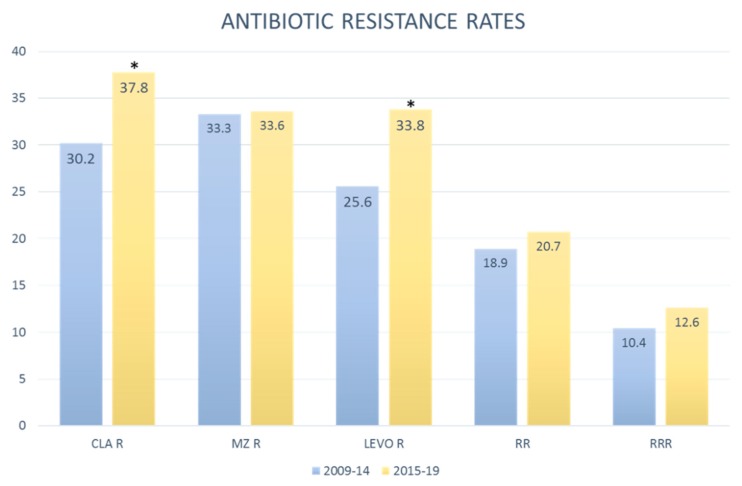
*H. pylori* antibiotic resistance rates in 2009–2014 and 2015–2019. (* Statistically significant). CLA: clarithromycin. Mz: metronidazole. Levo: levofloxacin. R: resistant. RR: resistant to clarithromycin and metronidazole. RRR: resistant to clarithromycin, metronidazole and levofloxacin.

Data collected from 2009 to 2014 were compared to data collected from 2015 to 2019 to analyze primary drug resistance trends (Figure 2). Antibiotic resistance rates in patients diagnosed in the first and second five-year period were, respectively, clarithromycin 30.2% (95% CI 27.2–33.3) and 37.8% (95% CI 34.2–41.4); metronidazole 33.3% (95% CI 30.2–36.5) and 33.6% (95% CI 30.2–37.1); levofloxacin 25.6% (95% CI 22.8–28.5) and 33.8% (95% IC 30.4–47.4). Double resistance clarithromycin-metronidazole 18.9% (95% CI 16.4–21.6) and 20.7% (95% CI 17.8–23.8) triple resistance clarithromycin-metronidazole-levofloxacin 10.4% (95% CI 8.5–12.5) and 12.6% (95% CI 10.3–15.2). 

Total resistance data are shown in Table 3 and Table 4 for 2009–2014 and 2015–2019, respectively.

Although past antibiotic abuse is a key factor for the increase in *H. pylori* antibiotic resistance [15], we investigated other factors. Patients diagnosed in the second five-year period (2014–2019) had a higher probability to be clarithromycin and levofloxacin resistant. Female gender was correlated to metronidazole and double (clarithromycin + metronidazole) resistance, with a subsequent high probability of treatment failure. Age was correlated with clarithromycin resistance (younger than 50 years old), levofloxacin and triple resistance (older than 50 years old). Active smokers had higher probability of being resistant to metronidazole. 

## 3. Discussion

The treatment of *H. pylori* infection has become complicated by the increasing trend in antimicrobial resistance worldwide [19,20] primarily because antibiotic agents used for *H. pylori* eradication are also widely used to treat other infections [7,8,12]. Antibiotic resistance develops continuously, so it is very important to carry out periodic assessments of *H. pylori* primary antibiotic resistance rates and to monitor the efficacy of first line treatments [13,14,15,16]. Aims of this study were to evaluate the prevalence of primary resistance to clarithromycin, metronidazole, and levofloxacin and to assess the effectiveness of sequential therapy over a 10 years period. The resistance rates to clarithromycin and levofloxacin had both a statistically significant increase (*p* < 0.05). Despite this, the general eradication rate of the sequential therapy was still optimal, being constantly higher than 90% (PP 93.4%, 95% CI 92–94.6; ITT 87.5%, 95% CI 85.7–89). Only in patients harboring resistant strains to both clarithromycin and metronidazole, the eradication rate was suboptimal (PP 83.6%, 95% CI 78.7–87.5; ITT 77%, 95% CI 71.8–81.5). Nevertheless, it is important to stress that eradication rates had a significant decrease in the second five-year period, going from PP 95.3% (95% CI 93.6–96.5) and ITT 91% (95% CI 88.9–92.7) to PP 90.4% (95% CI 87.6–92.8) and ITT 82.1% (95% CI 78.8–85.1) (*p* < 0.05). Data of patients of the second five period who took Pylera^®^ therapy have already been published in a previous ad hoc study [21]. We investigated the role of factors potentially related with bacterial resistance (Table 5a,b).

The correlation between the year of *H. pylori* infection diagnosis (2015–2019) with clarithromycin and levofloxacin resistance confirms the significant increase in these antibiotics resistance rates previously observed [17]. Isolates collected from women patients are more prone to metronidazole and double clarithromycin/metronidazole resistance; in fact, macrolides and nitroimidazoles are widely used to treat urinary tract infections in women, inducing a selective pressure on *H. pylori* strains [2,22]. Age was correlated with clarithromycin resistance (younger than 50 years old), levofloxacin, and triple resistance (older than 50 years old) [22], this could be due to patients being prescribed more antibiotics (especially fluoroquinolones) [23,24] at an older age. Smokers had higher probability to be infected by a strain resistant to metronidazole; the correlation between smoke and therapy failure has been described in literature [25,26], so this observation is very interesting to be further investigated.

## 4. Materials and Methods 

### 4.1. Patients

This was a retrospective, single-center study (Sant’Orsola Hospital, Bologna, Italy) evaluating consecutive Italian patients referred by their physicians to our unit for upper endoscopy. Naïve (never treated for *H. pylori*) *H. pylori* positive patients diagnosed from January 2009 to January 2019 were considered. The exclusion criteria were (1) age 18 years; (2) previous gastric surgery; (3) use of PPI or antibiotics in the 2 weeks before the endoscopy; (4) known allergy to macrolides, nitroimidazoles, or penicillins. Each patient provided us personal information such as age, weight, height, smoking habits, daily intake of alcohol, familiarity for gastric cancer, educational qualifications, and region of birth and residence. All participants provided written informed consent. The study was approved by the local Ethical Committee and performed according to guidelines for Good Clinical Practice [27] and the Declaration of Helsinki [28].

### 4.2. Endoscopy and H. pylori Assessment 

Prior to the endoscopy all patients were subjected to ^13^C-urea breath test to detect *H. pylori* infection. The ^13^C-UBT was performed after an overnight fast. A baseline breath sample was obtained, and 75 mg of ^13^C urea with citric acid (1.5 g) was administered as an aqueous solution. Another breath sample was collected 30 min after the test solution was administered. Breath samples were analyzed with non-dispersive infrared spectroscopy (HeliFAN Plus, Fischer Analytic Instruments, Germany). The results of the test were considered as positive if the difference between the baseline sample and the 30-min sample exceeded 4 parts per 1000 of ^13^CO_2_, according to the manufacturer’s instructions. During endoscopy biopsy specimens (two from the antrum, two from the corpus, one from incisura angularis) were taken for histology (haematoxylin-eosin for pathological assessment and Giemsa for *H. pylori* staining). One bioptic sample from the antrum was used for rapid urease test (RUT) as routinely performed in our center, and one additional antral biopsy was used for bacterial culture and drug-susceptibility test. *H. pylori* status was assessed through CRM (composite reference method: at least two tests positive or only culture positive). Treatment success was evaluated by using a standard ^13^C-urea breath test performed 6–8 weeks after treatment ended. In the event of an early interruption of eradication therapy, ^13^C-UBT was performed after at least 7 days of treatment. Patients undergoing therapy for fewer than 7 days were considered as drop-outs, and those who did not undergo ^13^C-UBT testing after treatment were considered as lost to follow-up evaluation. Based on endoscopic reports, for the purposes of the study, patients with either a peptic ulcer (ulceration 5 mm in diameter) or mucosal erosions (superficial lesion 4 mm) in the stomach or duodenum were grouped together as “peptic ulcer disease” (PUD). Non-ulcer dyspepsia was diagnosed when no macroscopic lesions were detected at endoscopy and patients were included in “non-ulcer disease” (NUD) group. 

### 4.3. Antibiotics Susceptibility Test for H. pylori

Biopsy specimens collected for bacterial culture were streaked immediately onto commercial selective medium Pylori Agar (BioMérieux Italia S.p.A., Italy). The plates were incubated under microaerobic conditions at 37 °C for 3–5 days. Once incubated, the colonies resembling *H. pylori* were identified by oxidase, catalase, and urease tests. Suspensions from the primary plates were prepared in sterile saline solution to McFarland opacity standard 4, approximately 10^9^ colony forming units (CFU)/mL to perform an E-Test (BioMérieux Italia S.p.A., Italy). A total of three agar plates for every *H. pylori* strain were streaked in three directions with a swab dipped into each bacterial suspension to produce a lawn of growth. Three E-Test strips (clarithromycin 0.016–256 ug/mL, metronidazole 0.016–256 ug/mL, and levofloxacin 0.008–32 ug/mL) were placed each onto a separate plate, which was incubated immediately in a microaerobic atmosphere at 37 °C for 72 h. A fourth plate was used as positive control. Clarithromycin, metronidazole, and levofloxacin resistance break points for the minimal inhibitory concentration (MIC) are greater than 0.5 mg/L, greater than 8 mg/L, and greater than 1 mg/L, respectively, according to the updated recommendations of the European Committee on Antimicrobial Susceptibility Testing (EUCAST) [29]. From 2015 Eucast established that clarithromycin MICs between >0.25 and ≤0.5 were to be considered as “indeterminate”, suggesting not to administer the drug in this case, so these strains were considered as “resistant” in this study [30]. Drug susceptibility test was not performed for amoxicillin and tetracycline because in Europe the resistance rate is lower than 1% [16].

### 4.4. Chemotherapy for H. pylori

According to Italian Guidelines and European Guidelines [8,18] the first line therapies were sequential or Pylera^®^ therapy. Sequential therapy consist of 5 days of a dual therapy with 40 mg PPI twice a day (before breakfast and dinner) and 1000 mg of amoxicillin twice a day (after breakfast and dinner); followed by 5 days of a triple therapy with 40 mg PPI twice a day (before breakfast and dinner) and clarithromycin 500 mg and metronidazole 500 mg both twice a day (after breakfast and dinner). From 2016 Pylera^®^ was available, a three in one capsule containing 140 mg bismuth subcitrate potassium, 125 mg metronidazole, and 125 mg tetracycline. Pylera^®^ therapy is therefore a 10 days quadruple therapy with 20 mg PPI twice a day (before breakfast and dinner) plus three Pylera^®^ capsules four times a day (after breakfast, lunch, dinner and before bedtime).

### 4.5. Potential Predictive Factors of Antibiotic Resistance 

Many factors were investigated as potentially correlated to resistance rates. Age (>50 years or ≤50 years), sex, BMI (>25 or ≤25), smoking habits (at least one cigarette a day), alcohol consumption (at least one glass a day), familiarity for gastric cancer (first degree relatives), educational level (until middle school or above middle school), City of residence, endoscopic findings (PUD or NUD), and year of *H. pylori* infection diagnosis (2009–2014 vs. 2015–2019). 

### 4.6. Statistical Analysis

Means and their 95% confidence intervals were calculated as suggested by Newcombe et al. [29]. Eradication rates were calculated both by intention-to-treat (ITT) analysis, including all the enrolled patients, and by per-protocol (PP) analysis, including patients who took more than 90% of their medications and completed follow-up evaluation. Comparisons among patient subgroups were performed using the Chi-square test (Yates correction when appropriate), odd ratio calculator. A *p* level less than 0.05 was considered significant. Statistical analysis was performed with MedCalc19.1.

## 5. Conclusions

The constant increase of resistance rates is a serious problem to be solved. Since virtually all *H. pylori* eradication regimens are based on antimicrobials used also for other infectious diseases, setting up regular monitoring of primary resistance for *H. pylori* (as well as for other microorganisms) should be considered. This would improve the use of appropriate antimicrobial agents and also it would provide an indirect indicator of their use (or abuse) in the population.

## Figures and Tables

**Table:** 

(a)						
Population Features	2009–2014	%	95% CI	2015–2019	%	95% CI
**Patients**	907			739		
**Male**	358	39.5	36.3–42.7	288	39.0	35.4–42.6
**Female**	549	60.0	57.3–63.7	451	61.0	57.4–64.6
**Age mean**	51.5			53		
**BMI mean**	24.3			25		
**Smokers**	205	22.6	19.9–25.5	158	21.4	18.5–24.5
**Alcohol**	135	14.9	12.6–17.4	99	13.4	11.0–16.1
**Cardioaspirin**	68	7.5	5.9–9.4	56	7.6	5.8–9.7
**Familiarity for gastric cancer**	118	13.0	10.9–15.4	89	12.0	9.8–14.6
**Compulsory education**	362	39.0	36.7–43.2	276	37.3	33.8–40.9
**High school**	362	39.9	36.7–43.2	311	42.1	38.5–45.7
**Graduation**	183	20.2	17.6–22.9	152	20.6	17.7–23.7
**Chief town**	397	43.8	40.5–47.1	284	38.4	34.9–42.0
**Emilia Romagna**	737	81.3	78.6–83.7	593	80.2	77.2–83.1

95% CI: 95% Confidence Interval. BMI: Body Mass Index.

**Table:** 

(b)			
Population Features	TOT	%	95% CI
**Patients**	1646		
**Male**	646	39.2	36.9–41.7
**Female**	1000	60.8	58.3–63.1
**Age mean**	52.3		
**BMI mean**	24.6		
**Smokers**	363	22.1	20.1–24.1
**Alcohol**	234	14.2	12.6–16.0
**Cardioaspirin**	124	7.5	6.3–8.9
**Familiarity for gastric cancer**	207	12.6	11.0–14.3
**Compulsory education**	638	38.8	36.4–41.2
**High school**	673	40.9	38.5–43.3
**Graduation**	335	20.4	18.4–22.4
**Chief town**	681	41.4	39.0–43.8
**Emilia Romagna**	1330	80.8	78.8–82.7

TOT: totals from 2009 to 2019. 95% CI: 95% Confidence Interval. BMI: Body Mass Index.

**Table 2:** Endoscopic reports.

UGIE Reports (2009–2019)	No.	%	95% CI
**NUD**	1413	85.8	84.0–87.4
**PUD**	204	12.3	10.8–14.0
**MALT lymphoma**	20	1.2	0.7–1.8
**Gastric cancer**	9	0.5	0.2–1.0

PUD: peptic ulcer (ulceration 5 mm in diameter) or mucosal erosions (superficial lesion 4 mm) in the stomach or duodenum. NUD: no macroscopic lesions detected. MALT: mucosal-associated lymphoid tissue.

**Table 3:** Primary resistance patterns in *H. pylori* strains collected in 2009–2014.

907 Naïve	No.	%	95% CI
ClaR, MzR, LevoR	94	10.4	8.5–12.5
ClaR, MzR, LevoS	77	8.5	6.8–10.5
ClaR, MzS, LevoR	31	3.4	2.3–4.8
ClaR, MzS, LevoS	72	7.9	6.3–9.9
ClaS, MzR, LevoR	46	5.1	3.7–6.7
ClaS, MzR, LevoS	85	9.4	7.6–11.5
ClaS, MzS, LevoR	61	6.7	52.0–8.6
ClaS, MzS, LevoS	441	48.6	45.3–51.9
Cla R tot	274	30.2	27.2–33.3
Cla S tot	633	69.8	66.7–72.8
Mz R tot	302	33.3	30.2–36.5
Mz S tot	605	66.7	63.5–69.8
Levo R tot	232	25.6	22.8–28.5
Levo S tot	675	74.4	71.5–77.2
ClaR, MzR tot	171	18.9	16.4–21.6

R: resistant. S: susceptible. Cla: clarithromycin. Mz: metronidazole. Levo: levofloxacin.

**Table 4:** Primary resistance patterns in *H. pylori* strains collected in 2015–2019.

739 Naive	No.	%	95% CI
ClaR, MzR, LevoR	93	12.6	10.3–15.2
ClaR, MzR, LevoS	60	8.1	6.3–10.3
ClaR, MzS, LevoR	46	6.2	4.6–8.2
ClaR, MzS, LevoS	80	10.8	8.7–13.3
ClaS, MzR, LevoR	46	6.2	4.6–8.2
ClaS, MzR, LevoS	49	6.6	4.9–8.7
ClaS, MzS, LevoR	65	8.8	6.9–11.1
ClaS, MzS, LevoS	300	40.6	37.0–44.2
Cla R tot	279	37.8	34.2–41.4
Cla S tot	460	62.2	58.6–65.8
Mz R tot	248	33.6	30.2–37.1
Mz S tot	491	66.4	62.9–69.8
Levo R tot	250	33.8	30.4–37.4
Levo S tot	489	66.2	62.6–69.6
ClaR, MzR tot	153	20.7	17.8–23.8

R: resistant. S: susceptible. Cla: clarithromycin. Mz: metronidazole. Levo: levofloxacin.

**Table:** 

(a)			
Variables	Patterns of Resistance	OR	*p* Value
**2015–2019**	Cla R	1.4	0.001 *
	Mz R	1.01	0.910
	Levo R	1.48	0.000 *
	ClaR, MzR	1.23	0.348
	ClaR, MzR, LevoR	1.24	0.158
**Sex female**	Cla R	1.22	0.069
	Mz R	1.78	0.000 *
	Levo R	0.99	0.927
	ClaR, MzR	1.47	0.003 *
	ClaR, MzR, LevoR	1.18	0.309
**Age > 50 years**	Cla R	0.79	0.027 **
	Mz R	1	0.7054
	Levo R	1.38	0.003 *
	ClaR, MzR	1	0.8291
	ClaR, MzR, LevoR	1.1	0.003 *
**BMI > 25**	Cla R	0.9	0.367
	Mz R	0.9	0.280
	Levo R	1.06	0.608
	ClaR, MzR	0.9	0.113
	ClaR, MzR, LevoR	0.88	0.401
**Smokers**	Cla R	1.1	0.447
	Mz R	1.87	0.000 *
	Levo R	1.01	0.927
	ClaR, MzR	0.97	0.828
	ClaR, MzR, LevoR	0.96	0.816

OR: Odd Ratio. BMI: Body Mass Index. * Statistically significant. ** protective.

**Table:** 

(b)			
Variables	Patterns of Resistance	OR	*p* Value
**Daily alcohol consumption**	Cla R	1.01	0.954
	Mz R	0.84	0.282
	Levo R	1.06	0.701
	ClaR, MzR	0.94	0.715
	ClaR, MzR, LevoR	1.02	0.725
**Familiarity for gastric cancer**	Cla R	0.94	0.689
	Mz R	0.95	0.733
	Levo R	0.93	0.699
	ClaR, MzR	0.94	0.744
	ClaR, MzR, LevoR	0.87	0.556
**Level of education** (**till middle school)**	Cla R	0.86	0.153
	Mz R	0.99	0.899
	Levo R	1.04	0.724
	ClaR, MzR	0.96	0.742
	ClaR, MzR, LevoR	1.04	0.809
**Residence (main city)**	Cla R	0.92	0.409
	Mz R	1	0.637
	Levo R	1.08	0.469
	ClaR, MzR	0.89	0.375
	ClaR, MzR, LevoR	1.04	0.797
**PUD**	Cla R	0.8	0.070
	Mz R	0.77	0.120
	Levo R	0.85	0.350
	ClaR, MzR	0.74	0.157
	ClaR, MzR, LevoR	0.78	0.380

OR: Odd Ratio. PUD: peptic ulcer disease.
